# Prevalence of Sjögren’s syndrome in the general adult population in Spain: estimating the proportion of undiagnosed cases

**DOI:** 10.1038/s41598-020-67462-z

**Published:** 2020-06-30

**Authors:** Javier Narváez, Simón Ángel Sánchez-Fernández, Daniel Seoane-Mato, Federico Díaz-González, Sagrario Bustabad

**Affiliations:** 1 Department of Rheumatology (Planta 10-2), Hospital Universitario de Bellvitge, Feixa Llarga, s/n, Hospitalet de Llobregat, 08907 Barcelona, Spain; 2 Rheumatology Unit, Complejo Hospitalario Mancha Centro, Alcázar de San Juan, Ciudad Real Spain; 3 Unidad de Investigación, Sociedad Española de Reumatología, Madrid, Spain; 4 Department of Rheumatology, Hospital Universitario de Canarias, La Laguna, Santa Cruz de Tenerife Spain

**Keywords:** Rheumatic diseases, Health policy, Epidemiology, Rheumatology

## Abstract

To estimate the prevalence of Sjögren’s syndrome (SS) in the adult Spanish population we performed a population-based multicenter cross-sectional study. Cases were defined by the American-European Consensus Group criteria proposed in 2002. A total of 4,916 subjects aged 20 years or over were included. The estimated prevalence of SS (including primary and secondary forms) in the adult population in Spain was 0.33% (95% CI 0.21–0.53). Extrapolating to the total population of the country aged ≥ 20 years (around 37.7 million persons), there would be around 125,000 cases of SS in Spain. Considering only primary SS, the estimated prevalence was 0.25% (95% CI 0.15–0.43) or 1 person in 400. The prevalence of primary SS in Spain is comparable to that reported in other European studies with a similar design and diagnostic criteria. Based on these results, primary SS could not be considered a rare (orphan) disease. Only 50% of cases had already been diagnosed with SS prior EPISER 2016 study, confirming the existence of a non-negligible proportion of undiagnosed cases in the general population.

## Introduction

Sjögren’s syndrome (SS) is a chronic systemic autoimmune disease that mainly targets the exocrine glands, leading to dryness of the main mucosal surfaces and occasional glandular enlargement. In addition, a variety of systemic or extraglandular manifestations may occur, including fatigue, musculoskeletal symptoms, cutaneous lesions and internal organ involvement (e.g., pulmonary, renal, hepatic, and neurologic)^1^. Disease severity can vary over a wide range^1^.

SS occurs in a primary form not associated with other diseases and a secondary form that complicates or overlaps with other rheumatic conditions, most commonly rheumatoid arthritis (RA), systemic lupus erythematosus (SLE) and systemic sclerosis. Primary SS (pSS) is also associated with an increased risk of non-Hodgkin’s lymphoma (the lifetime risk of complications in SSp is approximately 5%)^2^.

Although the past few years have witnessed major advances involving B-cell hyperactivity in the pathogenesis of pSS^1^, its etiology remains incompletely understood. The most frequently proposed hypothesis centers on the effects of multiple, mainly unknown, environmental factors affecting individuals with a specific genetic susceptibility^1^.

Evaluations of the incidence and prevalence of pSS vary widely^3–24^. Previous epidemiological studies have revealed discrepant prevalences, ranging from 0.01% to more than 3% of the general population, which has generated controversy about whether pSS should be considered a rare disease or not^3–26^. It is unclear if these differences in prevalence reflect a real variability between populations or methodological differences (different diagnostic criteria and study designs).

It is critical to determine the prevalence of pSS to better understand its etiopathogenesis (assuming the existence of different rates between populations with different genetic and environmental backgrounds), and to determine the burden of the disease. In an era of increasing medical costs, financial constraints and managed health care, we need reliable prevalence rates to help improve health care planning and disability compensation in countries with free and universal health coverage. Some studies have shown that health expenses of patients with pSS are double than those of the mean for primary care patients and similar to those of patients with RA^27,28^.

The prevalence of SS has never been evaluated in Spain. This observation prompted the Spanish Society of Rheumatology (SER) to include this disease in EPISER 2016, a population-based study to estimate the prevalence of rheumatic diseases in the adult population^29^. In addition to our study, we also performed a systematic review of epidemiologic studies of pSS that have evaluated its prevalence according to the 2002 revised American-European Consensus Group (AECG) criteria^30^ in order to compare their prevalence rates with our results. Using this approach, we hope to obtain a more reliable estimate of the variations in the prevalence of pSS.

## Methods

### Sample selection

The EPISER 2016 is an epidemiological study of the major rheumatic diseases (RA; SLE; symptomatic osteoartrhritis of the hand, knee, hip, cervical and lumbar spine; fibromyalgia; ankylosing spondylitis; psoriatic arthritis; Sjögren’s syndrome; gout; and symptomatic osteoporotic fracture) in the adult population in Spain undertaken by the Spanish Society of Rheumatology [Sociedad Española de Reumatología (SER)]. The rationale, methods, and general characteristics of the sample have been published previously^29,31^.

Briefly, the EPISER 2016 is a multicenter, cross-sectional study that included 78 municipalities randomly selected throughout the 17 Spanish autonomous communities. A representative sample of adults aged 20 years or older and residing in the selected municipalities was selected by multistage stratified random sampling. We used the cutoff of 20 years of age because in the Spanish national statistics institute (INE), the census data are for 5-years periods, with the first of adulthood being 20–24 years.

Stratification was done by sex, age (in blocks of 10 years) and the rural/urban nature of the municipalities, in accordance with the distribution of the population in Spain. As EPISER 2016 included various diseases, for sample size calculation we focused on RA and psoriatic arthritis prevalence (a choice driven by convenience, based on the previous EPISER 2000^32^. Assuming a Poisson distribution, a sample comprising 4,000 individuals would yield a 95% confidence interval (CI) of 0.30–0.77 for an expected prevalence of 0.5% (RA) and of 0.14–0.54 for an expected prevalence of 0.3% (psoriatic arthritis). Based on an assumed missing values rate of 20%, we deemed it necessary to include around 5,000 individuals.

### Ethics

The study was first approved by the Research Ethics Committee of Hospital Universitario de Canarias (Acta 12/2016) and subsequently by the ethics committees of all the participant hospitals. All methods carried out in accordance with relevant guidelines and regulations. Oral informed consent was obtained from all subjects during the initial telephone contact. Their agreement or regret was recorded. Additionally, written informed consent was obtained from all subjects who went to the participating centers for physical examination and complementary tests.

### Recruitment of the selected population

From November 2016 to October 2017, the participants were contacted by using random digit dialing and a Computer Assisted Telephone Interviewing system (CATI) to conduct a questionnaire for the screening of the diseases under study. The survey was mostly performed via landlines, but in order to facilitate access to younger patients and expand the registry, we incorporated mobile phones beginning in March 2017, which represent 20.3% of the final sample. This figure reflects the proportion of homes in Spain that relied solely on a mobile telephone connection. Both for the randomized selection of telephones in each municipality as well as for conducting the initial screening interviews, an external company specialized in sociological studies was contracted, with experience in the areas of health and call center services (Ipsos España)^31^.

In cases of non-answered phone calls, a minimum of 6 attempts were made at different time frames. If after these attempts there was no answer or the subject refused to participate, another phone number within the same municipality was randomly selected.

The survey was divided into the following blocks of questions (see Annex 1 in Supplementary Material): sociodemographic, general health status, anthropometric and lifestyle (smoking and alcohol intake) variables, rheumatic disease screening and variables related to the use of health resources for osteoarticular problems (medical appointments and medications).

### SS screening and confirmation of positive screening results

The screening comprised 2 complementary paths for all the participants. On the one hand, they were asked whether they had already been diagnosed with SS or Sicca syndrome. In addition, a screening was carried out based on symptoms related to ocular dryness and xerostomia (questions 45 to 52 in Annex 1).

If during the call center interview the participant mentioned having been diagnosed with SS or sicca syndrome, he or she was asked to identify the pertinent center and attending specialist. In those cases in which the individual was diagnosed at the referral hospital for the municipality, the consent of the patient was requested so that the rheumatologists who participated as researchers in that hospital could confirm the diagnosis by reviewing the clinical history. If the individual was attended at a different center that was not the referral hospital in the municipality and the clinical record was not available, the patient's permission was sought so that the researcher could contact his/her doctor.

Individuals who had not previously been diagnosed with SS, but who had a positive screening result based on symptoms, were contacted once again by phone to assess the clinical suspicion by means of a second, more specific questionnaire (see Annex 2 in Supplementary Material). This second telephone interview was carried out by the investigating rheumatologist from the referral hospital in the municipality.

In those cases in which a suspicion of SS persisted after the second call, the subjects were offered an appointment with the investigating rheumatologist for diagnostic confirmation (physical examination and ancillary tests), according to the 2002 AECG criteria^30^. These were applied to confirm those cases not diagnosed before the study. For previously diagnosed patients, no concerted effort was made to verify that they fulfilled these criteria based on their clinical history; clearly identifiable diagnoses were accepted regardless of the criteria applied.

Cases in which the subject completed the call center interview with a positive screening result for SS and the rheumatologist could not confirm or rule out the diagnosis were classified as missing.

### Statistical analysis

The prevalence of SS and the 95% confidence interval (CI) were calculated considering the sample design. The weights were calculated depending on the selection probability for each stage of the sampling, taking as a reference the distribution of the population in Spain in 2016 according to Continuous Register Statistics from the Spanish Institute of Statistics. This weighing was carried out taking into account age (decades), sex, and geographic origin [3 zones were defined: North (Galicia + Asturias + Cantabria + Basque Country + Navarre + La Rioja), Mediterranean and the Canary Islands (Catalonia + Valencian Community + Balearic Islands + Murcia + Andalusia + Canary Islands), and Center (Community of Madrid + Castile and León + Aragón + Castile-La Mancha + Extremadura)]; based on these characteristics each individual in the sample represented a certain number of individuals in the population (see Annex 4 in Supplementary Material).

Multivariate logistic regression was used to identify associations of sociodemographic, anthropometric and lifestyle variables with SS. Variable were included in the multivariate analysis when *p* < 0.2 in the bivariate analysis. Statistical significance was defined as *p* ≤ 0.05.

Finally, we also calculated the predictive positive value (PPV) for each of the questions of the screening questionnaire.

All analyses were performed using IBM SPSS Statistics v22.

### Systematic review of the literature

In addition to our study, a systematic literature review according to the PRISMA guidelines^33^ was conducted to identify all epidemiologic studies with data on the prevalence of pSS published up to December 2019.

Inclusion criteria for study selection (search filters) were as follows: (1) patients with primary pSS (those studies that included patients with secondary SS complicating other rheumatic conditions were excluded); (2) diagnosis of pSS according to the 2002 AECG criteria^30^; (3) studies including only adults (> 18 years old); (4) study design: population-based studies and population surveys that examined an entire geographic region or that used a clearly defined random or clustered sampling procedure were included; reports consisting of surveys or audits in hospitals or clinics were excluded; (5) studies that reported the prevalence of pSS; and (6) studies in English, French, Spanish or Portuguese. No limitations were placed on ethnicity or human subjects.

Searches were conducted in PubMed for the period between January 1980 and December 2019 using the strategies recommended by the Cochrane Handbook. The PubMed comprehensive search strategy included the Mesh terms and keywords ("Sjögren’s Syndrome"[Mesh]) and ("Epidemiology"[Mesh] or epidemiol* or "Prevalence"[Mesh] or Prevalenc*). The reference lists of relevant articles were also reviewed to identify additional reports.

One reviewer (JN) collected the information from the included studies using ad hoc standard forms. Articles that did not fulfil all the inclusion criteria or that had insufficient data were excluded.

## Results

### Prevalence of SS in Spain

**Figure 1:**
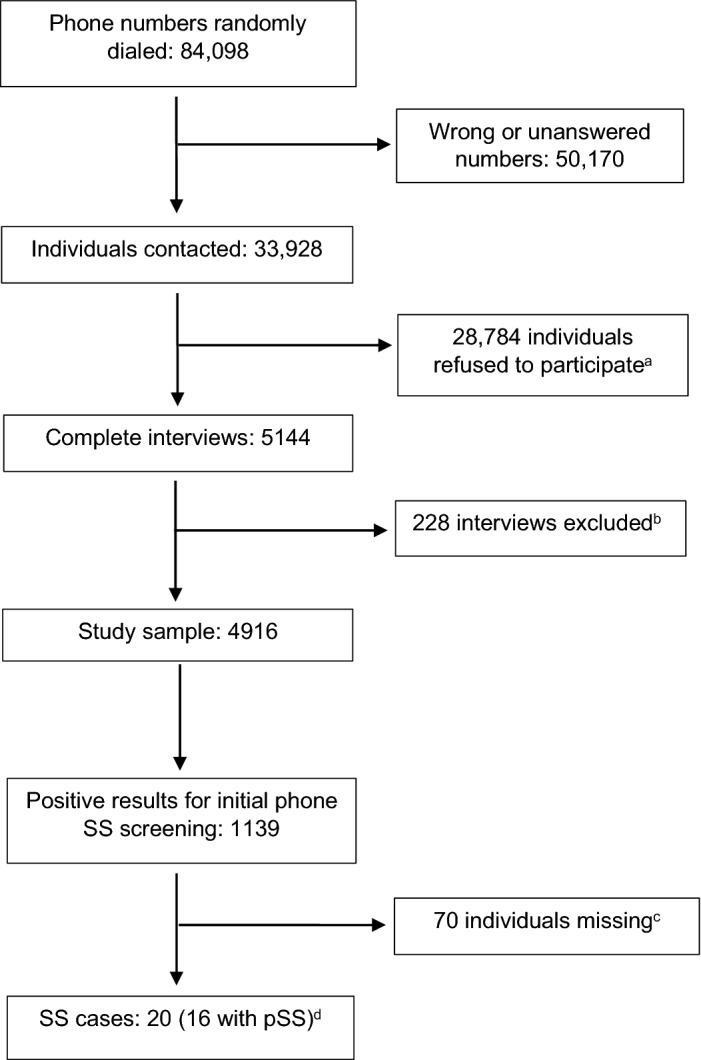
Flowchart showing participants in the study. ^a^27,892 refused in the very beginning of the phone call. ^b^Mainly after removal of duplicated interviews or excess numbers in certain sample strata. ^c^None of the 70 missing subjects had reported a previous diagnosis of SS or Sicca syndrome. ^d^Only 50% (10/20) of these cases had already been diagnosed with SS prior to the EPISER2016 study.

A detailed flow chart of participants in the study is depicted in Fig. 1. A total of 84,098 different phone numbers were dialed. Of these, 50,170 were wrong numbers or were unanswered; 28,784 individuals refused to participate (96.9% of them refused at the very beginning of the interview), and 5,144 complete interviews were completed. The response rate, once the individual had been contacted, was 15.2%.

The final sample comprised 4,916 individuals, mainly after removal of duplicate interviews or excess numbers in certain sample strata. Baseline characteristics of the whole sample and a comparison with the general population aged 20 years or older in Spain (reference population in EPISER2016) have been published in detail elsewhere^31^. For the purposes of this study, the simple can be considered representative of the population aged 20 years or over in Spain^31^.

A total of 1,139 subjects (22.8%) had a positive screening for SS (call center questionnaire*)*. A subsequent evaluation (review of their medical record and/or telephone call and medical consultation if needed) by a rheumatologist was possible in 1,069 subjects (none of the 70 missing subjects had reported a previous diagnosis of SS or Sicca syndrome).

Of these 1,069 subjects, 20 (1.87%) SS cases were detected, all of whom met the 2002 AECG criteria^30^ (see Annex 5 in Supplementary Material). Only 50% (10/20) of these cases had already been diagnosed with SS prior to the EPISER study. Four cases were patients with secondary SS complicating RA (3) or SLE (1); the remaining 16 were classified as pSS.

The estimated prevalence of SS (including primary and secondary forms) in the adult population in Spain was 0.33% (95% CI 0.21–0.53). Extrapolating to the entire population of the country aged 20 years or older (around 37.7 millions)^34^, there would be approximately 125,000 cases of SS in Spain.

Considering only pSS, the estimated prevalence of the disease was 0.25% (95% CI 0.15–0.43) or 1 person in 400.

### Predictive value of pSS screening

The negative predictive value (NPV) among the 1,542 participants with a positive screening result for the other diseases included in EPISER 2016 (negative for SS) was 100% (0 cases of SS were detected). Additionally, in a pre-planned substudy on 209 subjects randomly selected among those with a negative screening result for all the diseases included in EPISER 2016, no SS cases were detected.

The positive predictive value (PPV) of the screening questionnaire was 1.87% (20 cases among the 1,069 subjects with a positive screening result who completed the study).

Individual items of the screening questionnaire had a different PPV (see supplementary data 3). Reporting a previous diagnosis of SS or Sicca syndrome was the single best-discriminating item for SS (PPV 50%). Of the 6 questions related to oral and ocular symptoms none had a discriminative value by itself, with a PPV of less than 7% in all case.

Among the 3 questions that evaluated the presence of ocular dryness, the use of artificial tears 3 or more times per day was the one that had a slightly higher PPV (6.4%). The PPV was similar for the 3 questions that investigated the presence of xerostomia.

### Characteristics of the SS cases identified

The most important sociodemographic characteristics of the patients identified are shown in Table 1. Of the 20 patients, 17 were women (85%) and 3 (6%) were men, corresponding to a female-to-male ratio of 6:1. The mean age (standard deviation) of the subjects at the time of participation in the study was 57 (± 13.4) years. SS occurred more frequently between the fourth and fifth decades (50% of cases). Ninety five percent of the cases were subjects resident in urban municipalities.

**Table 1:** Variables associated with the presence of Sjögren’s syndrome (SS).

Variable	With SS (n = 20)	Without SS (n = 4826^a^)	Bivariate analyses	Multivariate analyses	
			*p* value	Odds ratio (95% CI)	p value
Age (years)			0.107		
20–39	2 (10%)	1,549 (32.1%)			
40–59	10 (50%)	1,856 (38.5%)		2,940 (0.627–13.776)	0.171
≥ 60	8 (40%)	1,421 (29.4%)		3.097 (0.642–14.948)	0.159
Gender (female)	17 (85%)	2,615 (54.2%)	**0.006**	3.967 (1.137–13.843)	**0.031**
Area of Spain			0.261		
North	9 (45%)	1,382 (28.6%)			
Center	4 (20%)	1,423 (29.5%)			
Mediterranean (+ Canary Islands)	7 (35%)	2,021 (41.9%)			
Level of education			0.752		
Elementary	9 (45%)	1,796 (37.2%)			
Middle	5 (25%)	1,258 (26.1%)			
High	6 (30%)	1,767 (36.6%)			
Body mass index			0.487		
Normal	12 (60%)	2,059 (44.4%)			
Underweight	0	54 (1.2%)			
Overweight	5 (25%)	1,847 (39.8%)			
Obese	3 (15%)	681 (14.7%))			
Tobacco			0.900		
Never	10 (50%)	2,373 (49.2%)			
Before	6 (30%)	1,294 (26.8%)			
Active smoking	4 (20%)	1,159 (24%)			
Birth abroad of Spain	2^b^ (10%)	336 (7%)	0.646		
Residence			0.062		
Rural	1 (5%)	1,098 (22.8%)		6.051 (0.809–42.285)	0.080
Urban	19 (95%)	3,728 (77.2%)			

^a^This number is the remainder of taking 20 cases with confirmed SS and 70 missing subjects from the 4,916 individuals of the final sample.

^b^One case in another European country and 1 case in South America.

### Literature review

**Figure 2:**
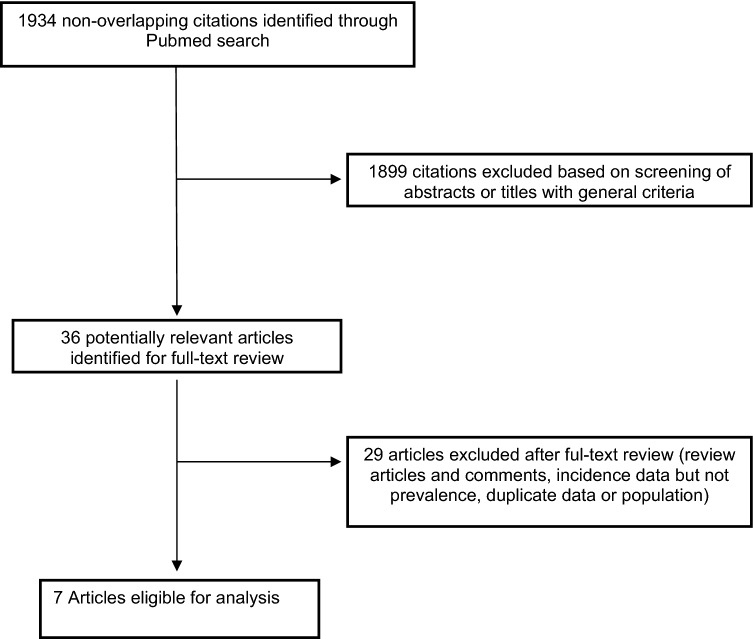
Flowchart showing article identification, inclusion and exclusion.

The PubMed search resulted in 1,934 non-overlapping article citations. A total of 1,899 articles were excluded based on the screening of abstracts or titles, leaving 36 articles for the full-text review and assessment for eligibility. Twenty-nine of these articles were excluded after retrieving the full-text articles, leaving 7 eligible studies for inclusion in the review^3,5,10,11,14–16^ (Fig. 2). The main characteristics of these studies are summarized in Table 2. In all cases, the diagnosis of pSS was made according to the 2002 AECG criteria^30^.

**Table 2:** Summary of studies on the prevalence of primary Sjögren’s syndrome in the adult population (≥ 18 years old) using the 2002 American-European Consensus Group (AECG) criteria.

Author^Ref^	Year	Country	Source of cases	Study design	Population size (N)	pSS cases (N)	Female/male (N)	Prevalence % Prevalence rate (95% CI)/100,000
Maciel et al^3^	2015	USA (Olmsted County, Minnesota)	Medical record linkage system	Population based	113,306	23	NS	0.02% 22 (1.3–3.1)
Valim et al.^5^	2013	Brazil (Vitoria)	Questionnaire Clinical examination	Cross-sectional population survey	1,205	2	NS	0.06% 60.82 (43.69–77.94)
Anagnostopoulos et al.^10^	2010	Greece (Prefecture)	Questionnaire Clinical examination	Cross-sectional population survey	1,705	4	NS	0.23% 234.60 (4.70–464.51)
Birlik et al.^11^	2009	Turkey (Balcova, Narlidere)	Questionnaire Clinical examination	Population survey	2,887	6	6/0	0.21% 207.83 (41.53–374.12)
Alamanos et al.^14^	2006	Greece (north-west Greece)	Personal registry physicians Medical record search	Population based	488,435	422	402/20	0.09% 86.40 (78.16–94.64)
Kabasakal^**a**^ et al.^15^	2006	Turkey (Bornova)	Questionnaire Clinical examination	Cross-sectional population survey	831	6	NS	0.72% 722.02 (144.29–1,299.75)
Trontzas et al.^16^	2005	Greece	Questionnaire Clinical examination	Population survey	8,470	13	NS	0.15% 0.08% ages 44–64 years 0.40% ages > 65 years 148.74 (67.89–229.60)

^a^The study was only conducted in female population.

All studies were performed between 2004 and 2017; five reported prevalence rates in European populations^10,11,14–16^ and two in the Americas (one in U.S. and one in South America)^3,5^. Various sources were used for case-finding purposes. In 5 studies, pSS cases were identified using questionnaires and clinical examination^5,10,11,15,16^. Other sources included physician registries and medical record databases^3,14^. One study only reported the prevalence of pSS in women^15^.

Although all the studies used the same diagnostic criteria, discrepancies were observed in prevalence figures, ranging from 0.02% of the general population to 0.72% Population size significantly affected the evaluation of prevalence in patients with pSS. In general terms, the prevalence rate reported in small studies was higher than that in larger studies. In addition, there were important differences depending on the study design and the case-finding methods employed, with population surveys based on sampling procedures and questionnaire-based surveys reporting higher prevalence rates than large population-based studies based on physician registries and medical record databases. These higher prevalences might stem in part from the fact that the tests required for disease classification according to the 2002 AECG criteria (ocular dryness objective assessments, salivary gland functional or morphologic tests, or salivary gland biopsies) are not always performed in clinical practice. This is reflected in the study by Maciel et al.^3^, based on manual medical records review, in which physician diagnoses yielded an age- and sex-adjusted pSS prevalence of 10.3 per 10,000 inhabitants, while according to AECG criteria, this prevalence was only 2.2 per 10,000^3^.

Taking into account these biases, in order to compare our results we focused only on those survey studies that evaluated the prevalence of pSS using a similar study design and case-finding method (sampling procedures and questionnaire based case-finding). Only five studies met these criteria (Table 2)^5,10,11,15,16^. Four were performed in Europe, though one of these was not comparable to ours as it only investigated the prevalence of pSS in women^15^. Of the remaining three, two were performed in Greece^10,16^ and reported a prevalence of 0.23% and 0.15%; the other one was conducted in Turkey and reported a prevalence of 0.21%^11^. The fifth was conducted in Brazil and reported a low prevalence compared to the European studies (0.06%), partly because it only included people aged 18–65^5^.

## Discussion

The prevalence of pSS had never been evaluated in Spain. Using a cross-sectional population study, we found that the prevalence of pSS in the general population of Spain was 0.25% (95% CI 0.15–0.43) or 1 person in 400. This prevalence is quite similar to those reported in other survey studies that have evaluated the prevalence of pSS in other European countries using a similar case-finding method and the same diagnostic criteria (0.15–0.23% in Greece and 0.21% in Turkey)^10,11,16^.

Based on these results, pSS should no longer be considered a “rare disease” in Spain. The European Commission on Public Health defines a rare disease as a condition with prevalence below 1 in 2,000 persons^35^. With this cut-off point, in the majority of studies conducted in Europe^10,11,14–16^ (regardless of the study design), pSS would similarly not be considered a *“*rare (orphan) disease” (Table 2). The question of whether pSS is rare or not has important consequences, not only for the public health system management of disease burden, but also for the development of future therapeutic agents for this indication. Indeed, the European Parliament and the Council adopted regulation (CE) No. 141/2000 on orphan drugs in 1999, which encourages the pharmaceutical and biotechnological industries to carry out research on and develop drugs to treat orphan diseases^26^.

In the USA a disease is considered rare when it affects less than 200,000 people nationwide^36^. Based on this cut-off point, in the two population-based study published to the date in U.S (the PR calculated in the study conducted by Izmirly et al. was 13.1 per 100,000 person-years)^3,24^ pSS should not be considered a rare disease in this country. Supporting this impression, a recent meta-analysis of epidemiological studies in primary SS, computed an overall prevalence of 60.82 per 100,000 inhabitants worldwide or 1 person in 1,644^25^. There are 11 studies from Europe, which gave a pooled prevalence rate of 71.22 cases per 100,000 inhabitants (one case per 1,404 persons).

The fact that the estimated prevalence rates vary between studies with similar design and diagnostic/classification criteria (from 0.01 to 0.23% in Europe and 0.02 to 0.06% in America)^4,9–11,14–17,24^ supports the potential existence of geographical variations in disease prevalence due to different genetic backgrounds or environmental factors, without ruling out a possible confounding role of healthcare differences between countries. In this sense, Izmirly et al*.* recently revealed differences in the incidence rates among Manhattan residents based on race/ethnicity in women, with Asian women ranking the highest^24^.

In our country the prevalence of pSS is about three times lower than RA (0.9% in EPISER 2016)^37^, but quite similar to SLE (0.21%)^38^. In this sense, it has been speculated that pSS could be the most common systemic autoimmune rheumatic disease (SARD) and the most underdiagnosed. Since usually it manifests with nonspecific symptoms like dry eyes, pain and fatigue, it is often overlooked and misdiagnosed for several years^39^. Results from the EPISER 2016 confirm the existence of a non-negligible percentage of undiagnosed pSS cases in our community (50% of the patients detected in our study had not been previously diagnosed). This fact could be important to explain why prevalence rates are lower in large population-based studies relying on physician registries and medical record databases, than in population surveys based on sampling procedures and questionnaire-based case-finding, even when performed in the same country^10, 15, 16^. Supporting this impression, in our study only a small minority of patients with sicca symptoms have SS according to the 2002 AECG criteria (less than 2%).

As expected, pSS in Spain is much more frequent in women than in men. Most of cases are recorded between the fourth and fifth decades of life, although its occurrence in patients aged 60 years or older was common (40% of cases). Our findings are in line with a previous Spanish study which also observed 14% of cases in patients older than 70 years^40^. In this sense, the prevalence of pSS in the elderly European population seems to be between five to eight times higher than that of the general population^12,16,19^. Several factors can contribute to this fact including a senile atrophy of the exocrine glands, higher rates of comorbidities such as diabetes and multidrug therapy, and the increased prevalence of autoantibodies documented in the elderly^41^. Finally, interestingly the prevalence of SS seems to be higher in urban areas than in rural settings, a trend that needs to be confirmed. This fact has been previously reported by Broten et al. (J. Rheumatol 2014;41:673–679) in a study that analyzed the prevalence of systemic autoimmune rheumatic diseases (including SLE, systemic sclerosis, pSS and polymyositis/dermatomyositis) across 7 Canadian provinces using population-based administrative data. After adjusting for demographics, they also observed a greater prevalence of SARDs in urban-versus-rural settings after adjusting for demographics^42^. Living in urban or rural areas can have an impact on environmental factors. So, depending on where you life, you are exposed to different environmental factors, which is of importance since currently potential environmental triggers (such as viruses) are thought to participate in pSS pathogenesis. However, when interpreting the results of our study, one needs to consider that EPISER 2016 was primary designed to estimate the global prevalence of several rheumatic diseases in Spain. The analysis of the association with sociodemographic, anthropometric and lifestyle variables was a secondary objective and, for pathologies with a low prevalence such as pSS, the statistical power for this analysis is limited.

During the design of EPISER 2016 study other alternatives were considered, but phone calls were the best option in our context for access to the general population. In Spain there are no administrative claims that allow reliable estimates of prevalence for the entire country. There could be other alternatives to phone calls for recruitment, such as postal mail, but the logistics were more complicated and a better response rate was not assured beforehand. In addition, the use of other different screening methods, such as identifying the cases directly from hospital/rheumatologists/electronic medical records, would not have allowed to detect undiagnosed cases.

Among the strengths of our study, it should be emphasized that criteria for SS symptomatic screening used in the first phone call allowed a very high sensitivity, similarly to what has been demonstrated in other rheumatic diseases also investigated in EPISER 2016^38,43,44^. Moreover, the number of missing values among subjects with positive screening was low (70/1,139).

Although a response rate of 15.2% can be interpreted as a possible source of bias, previous revisions examining this question have concluded that a low response rate does not necessarily implies a non-response bias on their own when the reasons for non-participating are unrelated to the variables of interest in the study^45–48,49^. As we have already stated in previous publications of EPISER 2016^38,43,44^, the response rate for calls in our study is similar to the most recent estimations for population-based telephone surveys and could be lower compared to other reports due to the demanding sampling requirements (strata based on rural/urban, sex and decades of age)^50,51^. These requirements, together with the similarity in the characteristics examined between the sample and the general population aged ≥ 20 years in Spain (reference population in EPISER 2016), as well as the weighting used in estimating the prevalence figures, indicate that the low response rate did not generate a significant bias invalidating the estimated prevalence^31,38,43,44^.

## Conclusions

Even using the same classification criteria for pSS (the 2002 AECG criteria)^30^, the prevalence rates in the studies that analyzed this issue are very much influenced by population size, case source, and study design. This makes it a challenge to determine the true prevalence of the disease.

The EPISER 2016 study is the first to report the prevalence of pSS in Spain: 0.25% (95% CI 0.15–0.43) or 1 person in 400. This prevalence is quite similar to that reported in other European studies with a similar design and diagnostic criteria (0.15–0.23% in Greece and 0.21% in Turkey)^10,11,16^. Thus, it could be inferred that the most reliable prevalence of pSS in the Mediterranean countries ranges between 0.20 and 0.25%.

Based on these results, SS cannot be considered a rare (orphan) disease. This study also confirms that a non-negligible proportion of pSS cases in the general population remain undiagnosed. Strategies for outreach and management of such undiagnosed cases are still necessary. Reliable prevalence rates should help improve the planning of health-care and disability compensation in national systems with universal coverage.

## Supplementary information

Supplementary information 1

Supplementary information 2

Supplementary information 3

Supplementary information 4

Supplementary information 5

## Data Availability

The authors confirm that all data underlying the findings are fully available without restriction. All relevant data are within the article.
