# Supplementary material for: Prevalence of Sjögren’s syndrome in the general adult population in Spain: estimating the proportion of undiagnosed cases
Source: Sci Rep. 2020 Jun 30;10:10627. doi: 10.1038/s41598-020-67462-z (PMC7327007; doi:10.1038/s41598-020-67462-z)
Supplement: Supplementary file 1 — Supplementary information 1 [file 41598_2020_67462_MOESM1_ESM.docx]

**Annex 1.**

**PREVALENCE OF SJÖGREN’S SYNDROME IN THE GENERAL ADULT POPULATION IN SPAIN: ESTIMATING THE PROPORTION OF UNDIAGNOSED CASES.**

Javier Narváez, Simón Ángel Sánchez-Fernández, Daniel Seoane-Mato, Federico Díaz-González, Sagrario Bustabad.

**Questionnaire used in the first phone call.**

Age__________ Sex____________

P1: Over the past 12 months, would you say that your state of health has been: very good, good, not very good, bad, or very bad?

P2: Over the past year, have you seen a doctor or made an appointment to see a doctor due to any bone or joint problems? If the answer is yes, ask question P2b: What specialization?: 1: emergencies; 2: general practitioner; 3:rheumatologist; 4: orthopedist; 5: physical rehabilitation doctor; 6: Internist; 7: neurosurgeon; 8: Others; 9: does not know; 99: has no reply. (Not mutually exclusive response options)

P3: Over the past year, have you taken any medications for 1 month or longer due to any bone or joint problems? If the answer is yes, ask question P3b: Do you remember the name of the medicine?

P4: Do you suffer from any rheumatic, bone, or joint disease?

P5: Has your doctor told you that you have any of the following diseases?

- Rheumatoid arthritis.

- Ankylosing spondylitis.

- Psoriatic arthritis.

- Lupus.

- Osteoarthritis/wear and tear of joints. If the answer is yes: In what part of your body?

- Fibromyalgia.

- Gout.

- Sjögren’s syndrome or Sicca syndrome.

P6: If the answer is yes to one of the diseases in P5, ask in what center and what specialization. Also, ask the patient if he/she could give the name of the doctor.

P7: As an adult, have you ever broken a bone without having a car accident or a severe blow? (***question for subjects ≥40 years old***). If the answer is yes, ask in what center he/she was attended so that this information can be confirmed later.

P8: Have you had a knee or hip replacement implant? (***question for subjects ≥40 years old***) (Response options: 0. No 1. hip 2. knee 3. hip and knee). If it is a hip replacement implant, ask whether it was due to osteoarthritis or due to fracture, and in what center to confirm this information. (*Note: if the subject says that he/she doesn´t have a hip replacement, but says that he has been operated on the hip, it will be considered an affirmative answer, as it could be because of a hip fracture.)*

P9: Do you feel pain in most of your body?

*If the answer to P9 is yes, ask P10*: Have you suffered from this pain for at least 3 months?

*If the answer to P10, ask P11:*

P11: Is this pain due to an accident, trauma, or blow?

P12: Do you suffer or have suffered from neck /cervical pain without having had a fall/ blow or overstrain? **(Ask if the patient ≥40 years old.)**

*If the answer to P12 is yes, ask P13*: Has that pain lasted for three consecutive months or more although the pain was not constant/ there were ups and downs?

*If the answer to P13 is yes, ask P14*: Does it get worse when moving your neck or turning your head?

P15: Do you have or have you had low back pain/pain in the waist/ back pain in kidney area / pain in lumbar region, without it being due to a fall/ blow or overstrain?

*If the answer to P15 is yes, ask P16*: Has that pain lasted for three consecutive months or more although the pain was not constant/ there were ups and downs?

*If the answer to P16 is yes, ask P17, P18, P19, P20, and P21:*

P17: Does it become worse when lifting weight or making a physical effort?

or

P18: Does it improve with physical activity and exercise?

P19: Does it improve with rest?

P20: Does the pain wake you up at night?

P21: Did it start before you were 45 years old?

P22: Does your heel hurt or did it hurt, or has it been swollen (without it being due to a blow or overstrain)?

P23: Do you have or have you had pain in any joints without it being due to a fall/ blow or overstrain?

*If the answer to P23 is yes, ask P24*: For more than 4 consecutive weeks?

*If the answer to P24 is yes, ask P25, P26, (P27+P28 or P30+31) (depending on the indicated area), P29 and P32***.**

P25: Have you ever had swelling or inflammation for more than 4 consecutive weeks of any of those painful joints?

P26: Specific area of the body: hands, hips/ groins, knees, ankles, feet. For each area, see whether it affects one side or two.

*If pain occurs in hips/ groins, knees, ankles or feet, ask:*

P27: Does it get worse when making a physical effort, walking, or going up/ down stairs?

or

P28: Does it improve with physical activity/ exercise?

P29: Does it improve with rest?

*If the pain is in hands, ask:*

P30: Does it get worse when using your hands or moving fingers?

or

P31: Does it improve when using your hands or moving fingers?

P29: Does it improve with rest?

*For all areas***:**

P32: Does it happen that in the morning you cannot move the affected area or you find it stiff?

*If the answer to P32 is yes, ask P33*: For more than half an hour?

*(Note: P32 and P33 are not taken into account for considering a positive screening).*

P34: Have you ever had pain **and** swelling of one or two joints which has completely disappeared in 1-2 weeks?

P35**:** Have you ever had pain **and** swelling of the big toe which has completely disappeared in 1-2 weeks?

P36: Do your fingers turn white or have color changes when it is cold *(remark that it must not be only cold hands or feet, they must also change color)*?

P37: Have you ever had ulcers/mouth sores for more than 2 consecutive weeks?

P38: Have you ever had been told that you had anemia or low leukocytes/white cells or low platelets? *(Note:* if the subject refers that a doctor has told him/her the cause, *consider the answer to P38 as “no”)*

P39: Have you ever had an eruption/rash on your cheeks or nose for at least 1 month?

P40: Do you get a skin eruption/ rash after being in the sun not for so long as to get burnt?

P41: Have you ever had pain for several days when breathing deeply?

P42: Have you ever been told that proteins appear in your urine (or have you noticed your urine with a lot of foam)?

P43: Have you ever shed large amounts of hair in a short period of time?

P44: Have you ever had a convulsion or an epileptic attack?

P45: Do you have eye dryness that bothers you daily? *(Note: if the subject comments that it began after myopia surgery, consider the answer to P45 as “no”)*

*If the answer to P45 is yes, ask P46*: For more than 3 months?

P47: Do you repeatedly have a gritty sensation in your eyes?

P48: Do you use lubricant eye drops more than 3 times per day?

P49: Do you experience dry mouth on a daily basis?

*If the answer to P49 is yes, ask P50*: For more than 3 months?

P51: When eating, do you drink frequently to help you swallow?

P52: As an adult, have you ever had swelling of the side of the face/ the same swelling as in mumps, in a repeated or maintained way?

*If the answer to P16 or P22 or P25 is yes, ask P53a and P53b:*

P53a: Has your doctor told you that you have or ever had psoriasis?

P53b: Has any close relative (grandparents, parents, siblings, or children) been diagnosed with psoriasis?

*If the answer to P16 is yes, ask P54a and P54b:*

P54a: Has your doctor told you that you have or ever had uveitis (your eye became red and painful, so you had to visit the eye doctor and it was not due to infection).

P54b: Has any close relative (grandparents, parents, siblings, or children) been diagnosed with ankylosing spondylitis?

**If the subject is ≥40 years old, ask questions from P55 to P62 *(questions to assess risk of osteoporotic fracture, based on FRAX questionnaire)*:**

P55: Did your father or mother have a hip fracture?

P56: Are you taking or have you taken corticosteroids/cortisone/prednisone?

*If the answer to P56 is yes, ask P57*: During more than 3 consecutive months?

P58: Are you taking insulin?

P59: Has your doctor told you that you have adult osteogenesis imperfecta?

P60: Has your doctor told you that you suffer from a severe liver disease?

**If the subject is a woman,** ask P61: Could you tell me if you had menopause before you were 45 years old?

P62: Could you tell me how many alcoholic beverages you drink per day (as an average)? *(Note: encode the response in drink units. If the subject says that he/she doesn´t drink on a daily basis, consider it as a negative answer).*

P63: Could you tell me how much you weigh approximately?

P64: And how tall are you approximately?

P65: Could you tell me if you smoke? (*Note: Code responses as 1. Yes, I smoke daily. 2. Yes, I smoke, but not daily. 3. I currently don’t smoke, but I have smoked in the past. 4. I don’t smoke or have never smoked regularly 9. No reply*)

P66: Could you tell me the highest level of education you have achieved? *(Code responses in accordance with CNED14-A)*

P67: What is or what was your last employment? *(Code responses in accordance with CNO-11).*

P68: What is your country of birth? *(Code responses as 1. Spain 2.European Union 3. Rest of Europe 4. Africa 5. North America 6. Central America and Caribbean 7. South America 8. Asia 9.Oceania 99. No reply)*

P69: What is your nationality? *(Code answers as 1. Spanish 2. From European Union 3. From rest of Europe 4. African 5. North American 6. Central American and Caribbean 7. South American 8. Asian 9. Oceanian 10. Stateless person 99. No reply)*
