# Supplementary material for: Prevalence of Sjögren’s syndrome in the general adult population in Spain: estimating the proportion of undiagnosed cases
Source: Sci Rep. 2020 Jun 30;10:10627. doi: 10.1038/s41598-020-67462-z (PMC7327007; doi:10.1038/s41598-020-67462-z)
Supplement: Supplementary file 2 — Supplementary information 2 [file 41598_2020_67462_MOESM2_ESM.docx]

**Annex 2.**

**PREVALENCE OF SJÖGREN’S SYNDROME IN THE GENERAL ADULT POPULATION IN SPAIN: ESTIMATING THE PROPORTION OF UNDIAGNOSED CASES.**

Javier Narváez, Simón Ángel Sánchez-Fernández, Daniel Seoane-Mato, Federico Díaz-González, Sagrario Bustabad.

**Sjögren’s syndrome screening on the basis of the associated symptoms. First phone call.**

It was considered positive if the subject stated, at least, one of the following:

- Having had a daily feeling of dry eyes for more than 3 months.

- Having a recurrent sensation of sand or gravel in the eyes.

- Use of tear substitutes more than 3 times a day.

- Having had a daily feeling of dry mouth for more than 3 months.

- Drinking frequently to aid in swallowing when eating.

- Having had recurrently or persistently swollen salivary glands as an adult.

**Criteria for the maintenance of suspicion by the rheumatologist in the second telephone interview.**

Suspicion was not ruled out if the criteria applied for the first screening were met and, moreover, the subject stated no to be taking any medication causative of dryness (anti-hypertensives, anti-allergic drugs, medication for depression/anxiety/ other psychiatric conditions). If these criteria were positive, the rheumatologist could reject the suspicion, but should record the reason and that reason was reviewed by study coordinators. If these criteria were negative, the rheumatologist could maintain the suspicion if he/she considered it appropriate.
