# Supplementary material for: Prevalence of Sjögren’s syndrome in the general adult population in Spain: estimating the proportion of undiagnosed cases
Source: Sci Rep. 2020 Jun 30;10:10627. doi: 10.1038/s41598-020-67462-z (PMC7327007; doi:10.1038/s41598-020-67462-z)
Supplement: Supplementary file 3 — Supplementary information 3 [file 41598_2020_67462_MOESM3_ESM.docx]

**Annex 3.**

**PREVALENCE OF SJÖGREN’S SYNDROME IN THE GENERAL ADULT POPULATION IN SPAIN: ESTIMATING THE PROPORTION OF UNDIAGNOSED CASES.**

Javier Narváez, Simón Ángel Sánchez-Fernández, Daniel Seoane-Mato, Federico Díaz-González, Sagrario Bustabad.

**Positive predictive value (PPV) of each question included in the initial screening questionnaire.**

1. - *Reporting a previous diagnosis of Sjögren’s syndrome* or Sicca syndrome. Number of individuals (number of positive responses): 12. PPV: 50%.

2. - *Daily symptoms of dry eyes for at least 3 months*. Number of individuals (number of positive responses): 487. PPV= 3.1%.

3. - *Foreign body sensation in the eyes*. Number of individuals (number of positive responses): 522. PPV= 2.3%.

4. - *Use of artificial tears 3 or more times per day*. Number of individuals (number of positive responses): 141. PPV= 6.4%.

5. - *Daily symptoms of dry mouth for at least 3 months*. Number of individuals (number of positive responses): 372. PPV= 2.7%.

6. - *Need for liquids to swallow dry foods*. Number of individuals (number of positive responses): 269. PPV= 2.2%.

7. - *Recurrent or persistently swollen salivary glands*. Number of individuals (number of positive responses): 68. PPV= 2.9%.

- Positivity of the 3 questions that investigate the possible presence of keratoconjunctivitis sicca (questions 2+3+4): Number of individuals (number of positive responses): 74. PPV= 5.4%.

- Positivity of the 2 two first questions that investigate the possible presence of xerostomia (questions 5+6). Number of individuals (number of positive responses): 97. PPV= 4.1%.

- Positivity of the 3 questions that investigate the possible presence of xerostomia (questions 5+6+7). Number of individuals (number of positive responses): 12. PPV= 8.3%

- Some (1 or more) of the screening questions that investigate the possible presence of keratoconjunctivitis sicca + any (1 or more) of the screening questions that investigate the possible presence of xerostomia (excluding the presence of recurrent or persistently swollen salivary glands). Number of individuals (number of positive responses): 250. PPV= 4%.

- Some (1 or more) of the screening questions that investigate the possible presence of keratoconjunctivitis sicca + any (1 or more) of the screening questions that investigate the possible presence of xerostomia. Number of individuals (number of positive responses): 263. PPV= 3.8%.
