# Supplementary material for: Prevalence of Sjögren’s syndrome in the general adult population in Spain: estimating the proportion of undiagnosed cases
Source: Sci Rep. 2020 Jun 30;10:10627. doi: 10.1038/s41598-020-67462-z (PMC7327007; doi:10.1038/s41598-020-67462-z)
Supplement: Supplementary file 4 — Supplementary information 4 [file 41598_2020_67462_MOESM4_ESM.docx]

**Annex 4.**

**PREVALENCE OF SJÖGREN’S SYNDROME IN THE GENERAL ADULT POPULATION IN SPAIN: ESTIMATING THE PROPORTION OF UNDIAGNOSED CASES.**

Javier Narváez, Simón Ángel Sánchez-Fernández, Daniel Seoane-Mato, Federico Díaz-González, Sagrario Bustabad.

Weighing process.

*Weighting.

Sex: 1= men; 2= women.

if (Grupo=1 & sex 2 = 1 & Age group = 1 ) WEIGHT_GroupCCAA = 2922.59016393443.

if (Grupo=1 & sex 2 = 1 & Age group = 2 ) WEIGHT_GroupCCAA = 2940.56544502618.

if (Grupo=1 & sex 2 = 1 & Age group= 3 ) WEIGHT_GroupCCAA = 4300.27857142857.

if (Grupo=1 & sex 2 = 1 & Age group = 4 ) WEIGHT_GroupCCAA = 7583.45070422535.

if (Grupo=1 & sex 2 = 1 & Age group = 5 ) WEIGHT_GroupCCAA = 6908.88888888889.

if (Grupo=1 & sex 2 = 1 & Age group = 6 ) WEIGHT_GroupCCAA = 8400.02857142857.

if (Grupo=1 & sex 2 = 1 & Age group = 7 ) WEIGHT_GroupCCAA = 5777.8.

if (Grupo=1 & sex 2 = 2 & Age group = 1 ) WEIGHT_GroupCCAA = 6193.28571428571.

if (Grupo=1 & sex 2 = 2 & Age group= 2 ) WEIGHT_GroupCCAA = 5544.

if (Grupo=1 & sex 2 = 2 & Age group = 3 ) WEIGHT_GroupCCAA = 3833.47741935484.

if (Grupo=1 & sex 2 = 2 & Age group = 4 ) WEIGHT_GroupCCAA = 3277.91764705882.

if (Grupo=1 & sex 2 = 2 & Age group = 5 ) WEIGHT_GroupCCAA = 3929.85833333333.

if (Grupo=1 & sex 2 = 2 & Age group = 6 ) WEIGHT_GroupCCAA = 3769.9175257732.

if (Grupo=1 & sex 2 = 2 & Age group = 7 ) WEIGHT_GroupCCAA = 6337.29310344828.

if (Grupo=2 & sex 2 = 1 & Age group = 1 ) WEIGHT_GroupCCAA = 7779.81818181818.

if (Grupo=2 & sex 2 = 1 & Age group = 2 ) WEIGHT_GroupCCAA = 8533.57142857143.

if (Grupo=2 & sex 2 = 1 & Age group = 3 ) WEIGHT_GroupCCAA = 10529.5049019608.

if (Grupo=2 & sex 2 = 1 & Age group = 4 ) WEIGHT_GroupCCAA = 18437.6630434783.

if (Grupo=2 & sex 2 = 1 & Age group = 5 ) WEIGHT_GroupCCAA = 14344.6395348837.

if (Grupo=2 & sex 2 = 1 & Age group = 6 ) WEIGHT_GroupCCAA = 11664.1857142857.

if (Grupo=2 & sex 2 = 1 & Age group = 7 ) WEIGHT_GroupCCAA = 11417.9512195122.

if (Grupo=2 & sex 2 = 2 & Age group = 1 ) WEIGHT_GroupCCAA = 15988.4269662921.

if (Grupo=2 & sex 2 = 2 & Age group = 2 ) WEIGHT_GroupCCAA = 12729.2151898734.

if (Grupo=2 & sex 2 = 2 & Age group = 3 ) WEIGHT_GroupCCAA = 8062.078125.

if (Grupo=2 & sex 2 = 2 & Age group = 4 ) WEIGHT_GroupCCAA = 6596.75954198473.

if (Grupo=2 & sex 2 = 2 & Age group = 5 ) WEIGHT_GroupCCAA = 7388.28729281768.

if (Grupo=2 & sex 2 = 2 & Age group = 6 ) WEIGHT_GroupCCAA = 7766.0859375.

if (Grupo=2 & sex 2 = 2 & Age group = 7 ) WEIGHT_GroupCCAA = 12701.125.

if (Grupo=3 & sexo2 = 1 & Age group = 1 ) WEIGHT_GroupCCAA = 6154.40677966102.

if (Grupo=3 & sexo2 = 1 & Age group = 2 ) WEIGHT_GroupCCAA = 6069.1387283237.

if (Grupo=3 & sexo2 = 1 & Age group = 3 ) WEIGHT_GroupCCAA = 10621.5533980583.

if (Grupo=3 & sexo2 = 1 & Age group= 4 ) WEIGHT_GroupCCAA = 13637.7611940299.

if (Grupo=3 & sexo2 = 1 & Age group = 5 ) WEIGHT_GroupCCAA = 6020.35779816514.

if (Grupo=3 & sexo2 = 1 & Age group = 6 ) WEIGHT_GroupCCAA = 10295.4545454545.

if (Grupo=3 & sexo2 = 1 & Age group = 7 ) WEIGHT_GroupCCAA = 7647.2380952381.

if (Grupo=3 & sexo2 = 2 & Age group = 1 ) WEIGHT_GroupCCAA = 14097.431372549.

if (Grupo=3 & sexo2 = 2 & Age group = 2 ) WEIGHT_GroupCCAA = 11307.4505494506.

if (Grupo=3 & sexo2 = 2 & Age group = 3 ) WEIGHT_GroupCCAA = 5496.1269035533.

if (Grupo=3 & sexo2 = 2 & Age group = 4 ) WEIGHT_GroupCCAA = 5379.06896551724.

if (Grupo=3 & sexo2 = 2 & Age group = 5 ) WEIGHT_GroupCCAA = 5762.64516129032.

if (Grupo=3 & sexo2 = 2 & Age group = 6 ) WEIGHT_GroupCCAA = 6385.09090909091.

if (Grupo=3 & sexo2 = 2 & Age group = 7 ) WEIGHT_GroupCCAA = 9159.5.

For Sjögren’s syndrome.

|  | | Estimate | 95% Confidence Interval | | Unweighted counts |
| --- | --- | --- | --- | --- | --- |
|  |  |  | Low | Upper |  |
| Population size | No | 36676389,731 | 36305321,694 | 37047457,767 | 4826 |
|  | Yes | 122717,730 | 64794,460 | 180641,001 | 20 |
|  | Total | 36799107,461 | 36432840,914 | 37165374,008 | 4846 |
| Percentage | No | 99,67% | 99,47% | 99,79% | 4826 |
|  | Yes | ,33% | ,21% | ,53% | 20 |
|  | Total | 100,0% | 100,0% | 100,0% | 4846 |

For primary Sjögren’s syndrome

|  | | Estimate | 95% Confidence Interval | | Unweighted counts |
| --- | --- | --- | --- | --- | --- |
|  |  |  | Low | Upper |  |
| Population size | No | 36705280,155 | 36335026,545 | 37075533,765 | 4830 |
|  | Yes | 93827,306 | 43836,539 | 143818,073 | 16 |
|  | Total | 36799107,461 | 36432840,914 | 37165374,008 | 4846 |
| Percentage | No | 99,75% | 99,57% | 99,85% | 4830 |
|  | Yes | ,25% | ,15% | ,43% | 16 |
|  | Total | 100,0% | 100,0% | 100,0% | 4846 |
