# Supplementary material for: Prevalence of Sjögren’s syndrome in the general adult population in Spain: estimating the proportion of undiagnosed cases
Source: Sci Rep. 2020 Jun 30;10:10627. doi: 10.1038/s41598-020-67462-z (PMC7327007; doi:10.1038/s41598-020-67462-z)
Supplement: Supplementary file 5 — Supplementary information 5 [file 41598_2020_67462_MOESM5_ESM.docx]

**Annex 5.**

**PREVALENCE OF SJÖGREN’S SYNDROME IN THE GENERAL ADULT POPULATION IN SPAIN: ESTIMATING THE PROPORTION OF UNDIAGNOSED CASES.**

Javier Narváez, Simón Ángel Sánchez-Fernández, Daniel Seoane-Mato, Federico Díaz-González, Sagrario Bustabad.

**Classification criteria components of the newly diagnosed cases of Sjogren’s síndrome^†^.**

|  | **Ocular symptoms^1^** | **Oral symptoms^2^** | **Ocular signs^3^** | **Histopathology^4^** | **Objective evidence of salivary gland involvement^5^** | **Autoantibodies^6^** |
| --- | --- | --- | --- | --- | --- | --- |
| **Case 1** | **+** | **+** | **+** | **ND** | **+** | **+ (Ro/SSA)** |
| **Case 2** | **+** | **+** | **+** | **ND** | **+** | **+ (Ro/SSA and La/SSB)** |
| **Case 3** | **+** | **+** | **+** | **ND** | **-** | **+ (Ro/SSA and La/SSB)** |
| **Case 4** | **+** | **+** | **+** | **ND** | **+** | **+ (Ro/SSA and La/SSB)** |
| **Case 5** | **+** | **+** | **+** | **ND** | **+** | **+ (Ro/SSA)** |
| **Case 6** | **+** | **+** | **+** | **ND** | **-** | **+ (Ro/SSA)** |
| **Case 7** | **+** | **+** | **+** | **ND** | **+** | **+ (Ro/SSA and La/SSB)** |
| **Case 8** | **+** | **+** | **+** | **+** | **+** | **-** |
| **Case 9** | **+** | **+** | **+** | **ND** | **+** | **+ (Ro/SSA)** |
| **Case 10** | **+** | **+** | **+** |  | **+** | **+ (Ro/SSA and La/SSB)** |

Abbreviation: ND = Not done.

*^†^ Vitali, C. et al. Classification criteria for Sjögren's syndrome: a revised version of the European criteria proposed by the American-European Consensus Group. Ann Rheum Dis 61, 554-558 (2002).*

^1^ Ocular symptoms: a positive response to at least one of the following questions: 1) have you had daily, persistent, troublesome dry eyes for more than 3 month?; 2) Do you have a recurrent sensation of sand or gravel in the eyes?, and 3) Do you use tear substitutes more than 3 times a day?

^2^  Oral symptoms: a positive response to at least one of the following questions: 1)  Have you had a daily feeling of dry mouth for more than 3 months?; 2) Have you had recurrently or persistently swollen salivary glands as an adult?, and 3)  Do you frequently drink liquids to aid in swallowing dry food?.

^3^ Ocular signs: objective evidence of ocular involvement defined as a positive result for at least one of the following two tests: 1) Schirmer's I test, performed without anaesthesia (≤5 mm in 5 minutes), and 2) Rose bengal score or other ocular dye score (≥4 according to van Bijsterveld's scoring system).

^4^ Histopathology: In minor salivary glands (obtained through normal-appearing mucosa) focal lymphocytic sialoadenitis, evaluated by an expert histopathologist, with a focus score ≥1, defined as a number of lymphocytic foci (which are adjacent to normal-appearing mucous acini and contain more than 50 lymphocytes) per 4 mm^2^ of glandular tissue.

^5^ Salivary gland involvement: objective evidence of salivary gland involvement defined by a positive result for at least one of the following diagnostic tests: 1) Unstimulated whole salivary flow (≤1.5 ml in 15 minutes); 2) Parotid sialography showing the presence of diffuse sialectasias (punctate, cavitary or destructive pattern), without evidence of obstruction in the major ducts, and 3) Salivary scintigraphy showing delayed uptake, reduced concentration and/or delayed excretion of tracer.

^6^ Autoantibodies: presence in the serum of the following autoantibodies: Antibodies to Ro(SSA) or La(SSB) antigens, or both.
